# From Participation to Evidence: Listening‐Informed Policies and the Epistemic Role of Lived Experience in Population Health

**DOI:** 10.1111/hex.70696

**Published:** 2026-05-22

**Authors:** Serena Barello, Michela Monaci, Sarah Bigi, Beatrice Credi, Marko Korenjak

**Affiliations:** ^1^ WHYpsy Lab, Department of Brain and Behavioral Sciences University of Pavia Pavia Italy; ^2^ Department of Linguistic Sciences and Foreign Literatures Catholic University of the Sacred Heart Milan Italy; ^3^ European Liver Patients' Association (ELPA) Brussels Belgium; ^4^ Faculty of Government and European Studies New University Kranj Slovenia

## Abstract

Health policies and public services are increasingly expected to be evidence‐based, equitable and accountable at the population level. Yet the forms of evidence most commonly prioritised in policy‐making often fall short of capturing how health risks, services and prevention strategies are experienced and navigated across social groups and communities. This gap contributes to persistent mismatches between policy intent and lived social realities, with tangible consequences for population health outcomes, equity and public trust. Drawing on democratic accounts of policy legitimacy, scholarship on civil society as a knowledge intermediary and emerging regulatory recognition of experiential evidence, we propose Listening‐Informed Policies (LIP) as a governance‐oriented approach to population health decision‐making. We argue that listening constitutes a methodologically grounded epistemic practice oriented toward collective knowledge production. When institutionalised, listening can inform population‐level problem definition, policy design, implementation and evaluation, rather than serving merely as a legitimacy‐enhancing exercise. Using liver health as an illustrative context, we outline the core principles of LIP and consider their relevance for the prevention of non‐communicable diseases. Embedding experiential knowledge alongside biomedical and economic evidence—through organised civil society acting as an institutional intermediary—can strengthen population health governance on three complementary grounds: (i) democratically, by enhancing the legitimacy of decisions through the inclusion of affected populations; (ii) epistemically, by rendering visible social mechanisms and constraints not captured by conventional indicators, and (iii) pragmatically, by improving the alignment between policy design and real‐world implementation. LIP integrate these dimensions by positioning listening as a structured form of population‐relevant evidence.

**Public and Patient Contribution:** This viewpoint article was developed in collaboration with representatives and stakeholders engaged in the liver disease community, including perspectives emerging from patient advocacy and public engagement initiatives promoted within the European liver health landscape. The reflections presented in this paper were informed by ongoing dialogue with people living with liver conditions, patient advocates, clinicians, and researchers involved in initiatives aimed at strengthening meaningful patient involvement, communication, and advocacy in hepatology. These experiences contributed to shaping the focus and critical reflections discussed throughout the viewpoint.

## 1 Why Conventional Evidence Is Insufficient for Population Health Decision‐Making

Across health systems, population health policies are predominantly informed by epidemiological indicators, clinical outcomes and economic modelling. These forms of evidence are indispensable for surveillance, planning and accountability. At the same time, they offer only limited insight into how health risks are perceived, interpreted and acted upon across different social contexts, and how people collectively encounter prevention strategies and care pathways in everyday life. As a result, policies that appear technically sound may generate uneven uptake, delayed access or unintended inequities at the population level.

Liver health offers a particularly telling illustration of this gap. Despite established clinical pathways and growing policy attention, many liver conditions continue to be diagnosed at advanced stages. This pattern cannot be fully explained by biomedical indicators alone. Silent disease progression, stigma embedded in public narratives, uneven health literacy and structural barriers within primary care shape population‐level patterns of help‐seeking and diagnosis in ways that remain largely invisible to routine policy data. The consequence is a recurring misalignment between policy design and social realities, with implications for effectiveness, equity and trust in public institutions [1].

Importantly, these dynamics are not confined to liver health. Similar patterns can be observed across non‐communicable diseases, where prevention and early intervention depend not only on service availability but also on how communities understand and perceive risk, relate to institutions and navigate increasingly complex care systems.

## 2 Listening as Population‐Relevant Policy Evidence

This Viewpoint advances the argument that listening to lived experience should be recognised as a form of population‐relevant policy evidence, rather than treated as a supplementary or purely consultative activity. From the perspective of democratic governance, listening is not simply a communicative practice, but a mechanism through which collective needs, priorities and blind spots are rendered visible to institutions and incorporated into policy decision‐making. Democratic accounts of policy legitimacy emphasise that public decisions derive part of their validity from the inclusion of those affected in shaping norms and priorities, rather than merely responding to predefined choices [2].

## 3 Epistemic Status of Lived Experience

The recognition of lived experience as evidence rests on its capacity to generate systematically interpretable knowledge about how health systems, risks and interventions are encountered in practice. Experiential knowledge does not simply express individual opinion; when collected and analysed across populations, it reveals recurring patterns of meaning, barriers and adaptive behaviours that shape health outcomes. In this sense, it can illuminate (i) structural determinants that are not captured by routine data (e.g., stigma, institutional mistrust or administrative complexity), (ii) mechanisms underlying implementation failures (e.g., why services are not accessed or adhered to) and (iii) the ways in which policy problems are socially constructed and understood. These features justify its treatment as population‐relevant evidence, particularly when generated through systematic and transparent methods. These features justify its treatment as population‐relevant evidence, particularly when generated through systematic and transparent methods, as increasingly recognised in emerging policy and governance scholarship [3].

Organised civil society plays a crucial intermediary role in enabling such processes by collecting, aggregating and interpreting lived experience in ways that make it usable within policy and regulatory arenas [4]. Through this intermediary function, dispersed individual experiences are transformed into collective knowledge that can inform population‐level deliberation. Participatory governance scholarship further distinguishes these practices from episodic consultation, highlighting their capacity to reshape how policy problems are defined and how epistemic authority is distributed within decision‐making systems [5].

Against this background, it becomes clear that the form of listening proposed here differs both conceptually and methodologically from conventional consultation. Consultation typically occurs at discrete moments, is bounded by predefined agendas and treats experiential input primarily as feedback on policy options that have already been framed elsewhere. While such processes may enhance transparency or procedural legitimacy, they rarely alter underlying assumptions about population risk, care pathways or system priorities.

The conceptual differences between consultation and listening, and their implications for population health governance, are summarised in Table 1. By contrast, listening, as conceptualised within the Listening‐Informed Policies (LIP) framework, is an epistemic practice oriented toward collective knowledge production. It precedes decision‐making and focuses on understanding how policies are experienced and enacted across populations and social groups. Recent developments in regulatory science increasingly recognise experiential data as relevant to regulatory and policy decisions, reinforcing the institutional legitimacy of listening alongside biomedical and economic evidence [6]. In this sense, listening is not an adjunct to policy‐making, but an institutionalised mode of inquiry that complements existing evidence systems.

**Table 1:** Consultation versus listening in population health policy.

Dimension	Consultation of civil society	Listening‐informed policies (LIP)
Primary purpose	Transparency and stakeholder feedback	Collective knowledge generation for population health decisions
Position in the policy cycle	Episodic and downstream	Upstream and continuous across the policy cycle
Status of lived experience	Opinion or stakeholder perspective	Population‐relevant evidence
Unit of analysis	Individual or organisational viewpoints	Social patterns, shared mechanisms and community experiences
Role of civil society	Consultees or advocacy stakeholders	Epistemic intermediaries translating lived realities into evidence
Contribution to problem definition	Limited; problems largely pre‐framed	Substantive; reshapes understanding of population needs and risks
Governance function	Procedural legitimacy	Evidence‐informed governance
Implications for population health	Marginal adjustments to interventions	Structural alignment of policies with lived social realities

The argument advanced in this Viewpoint rests on three complementary but analytically distinct grounds. First, from a democratic perspective, listening enhances the legitimacy of population health decisions by incorporating the voices of those affected into the formation of collective priorities. Second, from an epistemic perspective, it expands what counts as relevant evidence by revealing social mechanisms, interpretive frames and structural constraints that remain invisible to conventional indicators. Third, from a pragmatic perspective, it contributes to more effective policy design and implementation by aligning interventions with how they are actually experienced and navigated across populations. While these justifications are mutually reinforcing, the central claim of LIP is primarily epistemic: that lived experience constitutes a distinct and necessary form of population‐relevant evidence for health policy.

While Table 1 outlines the conceptual distinction between consultation and listening, Table 2 provides an illustrative case from liver health, showing how listening‐informed approaches can reshape problem definition and inform population‐relevant policy action in practice.

**Table 2:** An illustrative case from liver health: population‐level implications of listening versus consultation.

Dimension	Consultation‐based approach	Listening‐informed approach
Policy objective	Improve early diagnosis of liver disease	Same objective, addressed through population‐level understanding
Mode of engagement	Episodic consultation on campaigns and guidelines	Sustained listening coordinated by civil society organisations
Focus of input	Acceptability of messages and materials	Collective patterns of uncertainty, stigma and pathway failures
Problem framing	Low awareness among individuals	Misalignment between public narratives, stigma and care pathways
Visibility of mechanisms	Limited	High: Social and organisational determinants become visible
Policy response	Refinement of awareness campaigns	Reorientation toward primary care practices and integrated prevention
Population health implications	Incremental change	Plausible potential for earlier detection, improved equity and stigma reduction, contingent on implementation

LIP are conceptually adjacent to, but distinct from, established approaches such as patient and public involvement (PPI), co‐production, deliberative governance and participatory health research. While these frameworks emphasise inclusion, collaboration and shared decision‐making, LIP specifically advance an epistemic and governance‐oriented shift: it positions listening not primarily as a participatory process, but as a structured mode of evidence production for population health decision‐making. In this sense, LIP complement rather than replace these approaches by clarifying how the outputs of participation—namely lived experience—can be systematically generated, interpreted and institutionalised as policy‐relevant evidence. This distinction is critical to avoid reducing listening to a procedural exercise and to foreground its role within evidence‐based governance, particularly in light of recent work highlighting the need to better integrate participatory processes with evidence production in policy systems [3, 7, 8].

## 4 Listening‐Informed Policies as a Governance Approach

**Figure 1:**
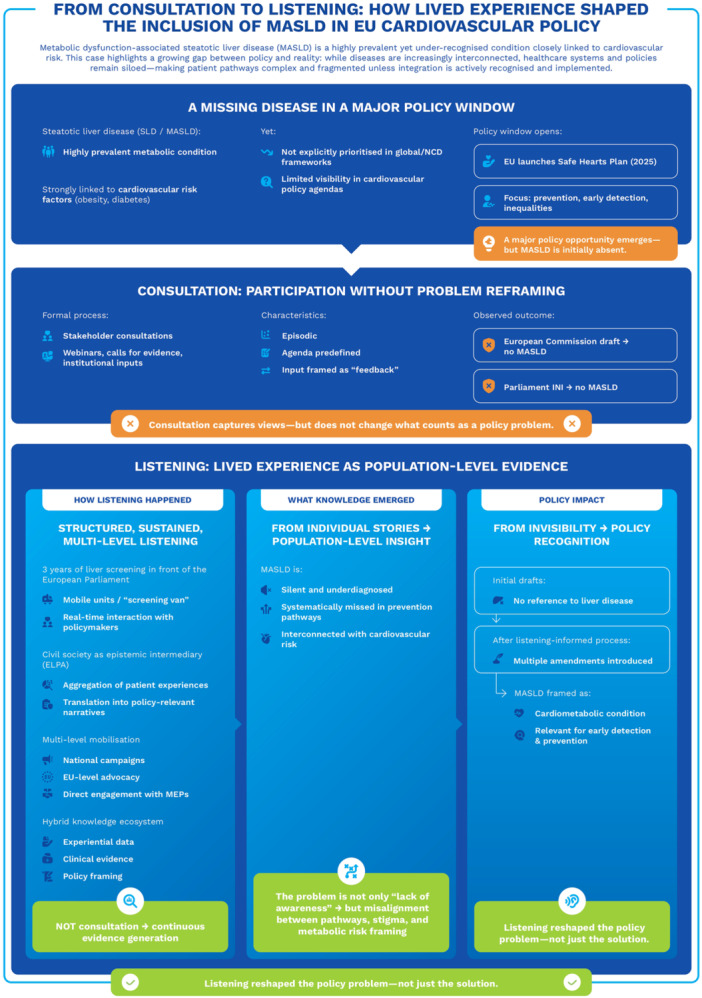
From consultation to listening: reframing MASLD within EU cardiovascular policy.

**Figure 2:**
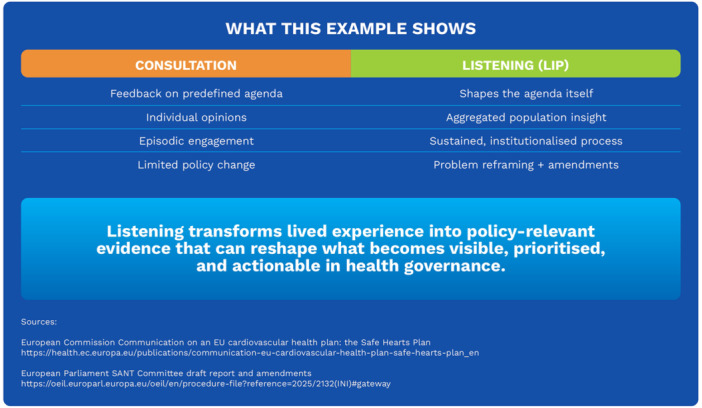
From consultation to listening‐informed policy‐making: key differences and lessons learned.

While not designed as a formal evaluative case, this example illustrates how sustained and structured engagement with lived experience can contribute to reframing policy priorities within existing health strategies (see Figure 1). The inclusion of metabolic dysfunction‐associated steatotic liver disease (MASLD) within the policy discourse surrounding the EU Safe Hearts Plan illustrates the distinction between consultation and listening in practice. While early stages of the policy process relied on standard consultation mechanisms that did not identify liver disease as a priority, sustained listening processes led by civil society organisations, specifically patient organisations, enabled the aggregation of lived experience into population‐level insight. Through continuous engagement, including community‐based screening initiatives, multi‐level advocacy and direct interaction with policymakers, experiential knowledge contributed to reframing MASLD as a cardiometabolic condition relevant to prevention and early detection. This example highlights how listening, as an epistemic practice, might reshape the definition of policy problems and open new avenues for integration into existing health strategies (see Figure 2).

The figure contrasts consultation with a listening‐informed approach in the EU Safe Hearts Plan. While consultation did not identify MASLD as a priority, sustained listening led by civil society (e.g., European Liver Patients' Association [ELPA]) enabled the translation of lived experience into population‐level insight, supporting its reframing as a cardiometabolic condition and its inclusion in the policy agenda

The figure contrasts consultation and LIP across four dimensions: agenda setting, type of knowledge generated, temporality of engagement and policy impact. While consultation relies on episodic input on predefined agendas and produces individual‐level opinions with limited influence on policy change, LIP are characterised by sustained, institutionalised processes that aggregate lived experience into population‐level insight, contributing to problem reframing and policy amendments.

## 5 From Experiential Input to Policy‐Relevant Evidence: Methodological Considerations

LIP should be understood as a governance‐oriented approach to population health evidence, rather than as a model of stakeholder engagement. Its core proposition is that collective lived experience requires dedicated structures, methods and interpretive processes in order to become policy‐relevant.

First, experiential knowledge should be generated through systematic and transparent approaches capable of identifying shared patterns across populations. These may include qualitative methodologies (e.g., in‐depth interviews, focus groups), participatory and co‐design processes, analysis of patient‐reported experience data and structured engagement platforms coordinated by civil society organisations. Second, such data require explicit strategies for aggregation and interpretation, moving from individual narratives to collective patterns while addressing issues of representation, bias and divergence across groups. Third, experiential evidence should be integrated with existing data systems, enabling triangulation with epidemiological and economic indicators. While a full methodological framework is beyond the scope of this Viewpoint, existing work in participatory health research, deliberative governance and the integration of lived experience into policy evidence provides a foundation for operationalising these principles [3, 7, 8].

Developed in collaboration with the ELPA, the LIP approach is directly relevant to liver health and transferable to other non‐communicable disease contexts.

## 6 Implications for Health Systems and Prevention Policy

The challenges illustrated through liver health (i.e., late diagnosis, fragmented pathways, stigma and unequal access) are echoed across many areas of prevention and chronic care. In each case, listening helps explain why technically sound policies may fail to achieve their intended population impact.

When listening is institutionalised, health systems signal responsiveness and accountability, reinforcing trust in public institutions [1]. As illustrated in Table 2, such shifts may contribute to earlier detection, improved equity and stigma reduction at the population level, particularly when experiential evidence is systematically integrated into policy design and implementation processes. LIP can support prevention strategies by aligning interventions with how risks and services are collectively experienced, identifying inequities invisible to routine indicators and anticipating implementation failures before they become systemic.

## 7 Conclusion

This Viewpoint argues that strengthening population health policy requires rethinking what counts as evidence, and how different forms of knowledge are generated, validated and integrated within governance processes. Liver health illustrates how reliance on conventional indicators alone can obscure critical social and organisational determinants of prevention and early detection. LIP offer a practical orientation for embedding collective lived experience within health policy and public service decision‐making.

Rather than calling for more consultation, LIP advocate for the institutionalisation of listening as a methodologically grounded form of population‐relevant evidence. By doing so, health systems can develop policies that are not only evidence‐based but also more equitable, implementable and responsive to social realities. From the standpoint of organised civil society, LIP therefore represent both a methodological innovation in how evidence is produced and a democratic imperative in how population health decisions are made.

## Author Contributions

**Serena Barello:** conceptualization, funding acquisition, writing – original draft, methodology, writing – review and editing, project administration, supervision. **Michela Monaci:** writing – review and editing. **Sarah Bigi:** conceptualization, writing – review and editing. **Beatrice Credi:** validation, writing – review and editing. **Marko Korenjak:** validation, writing – review and editing.

## Conflicts of Interest

The authors declare no conflicts of interest.

## Public and Civil Society Involvement

Civil society organisations and community representatives were actively involved in the conceptual development of the Listening‐Informed Policies approach. Members of European liver health organisations contributed to identifying systemic gaps in existing evidence frameworks and reflecting on the implications of listening for population health, prevention policy and public service decision‐making. As a conceptual Viewpoint, this article does not report primary data collection.

## Role of Civil Society Organisations

Representatives of civil society organisations contributed to the conceptual development of the Listening‐Informed Policies framework. No civil society organisation had any role in data collection, data analysis or interpretation, as this article does not report original empirical research.

## Data Availability

Data sharing is not applicable to this article as no datasets were generated or analysed during the current study.
